# Research Progress on 35S rDNA and 5S rDNA in Sugarcane: Challenges and Prospects

**DOI:** 10.3390/ijms26188773

**Published:** 2025-09-09

**Authors:** Xueting Li, Yirong Guo, Zhejun Guo, Nannan Zhang, Yawen Lei, Enping Cai, Zuhu Deng, Jiayun Wu

**Affiliations:** 1 Guangdong Sugarcane Genetic Improvement Engineering Center, Institute of Nanfan & Seed Industry, Guangdong Academy of Sciences, Guangzhou 510316, China; lxt19910226@163.com (X.L.); guoyirong0127@163.com (Y.G.); zhangnn23@mail2.sysu.edu.cn (N.Z.); leiyw@mail2.sysu.edu.cn (Y.L.); dlcep@foxmail.com (E.C.); 2 National Engineering Research Center for Sugarcane, Fujian Agriculture and Forestry University, Fuzhou 350002, China; 18350578110@163.com

**Keywords:** 35S rDNA, 5S rDNA, sugarcane, FISH, chromosome

## Abstract

rDNA is abundant in various organisms, typically expressed as conserved tandem repeats. It plays a crucial role in ribosome synthesis, gene transcription, and expression, and it affects the occurrence of diseases in both animals and plants, aging, protein synthesis, genomic stability, and genome evolution across a wide range of organisms. Among the different types of rDNA, 35S rDNA (also referred to as 45S rDNA) and 5S rDNA are particularly important in plant research. The use of 35S rDNA and 5S rDNA as probes has enabled the study of chromosomal composition, revealing species characteristics that are valuable for crop breeding, evolutionary biology, systematics, and other fields. This review focuses on the application of 35S rDNA and 5S rDNA and discusses research findings on sugarcane and its related germplasm that have been obtained through fluorescence in situ hybridization. This information has provided a foundation for understanding the genetic relationships, genetics, breeding, and evolutionary classification of sugarcane.

## 1. Introduction

### 1.1. Composition and Characteristics of 35S rDNA and 5S rDNA

Ribosomes are primarily composed of proteins and ribosomal RNA (rRNA), serving as the main site for protein synthesis in plants. The genes encoding rRNA are referred to as rDNA [1]. rDNA consists of highly conserved tandem repeat sequences that are widely present in the plant genome [2,3]. Repeat sequences are defined as identical DNA fragments that can occur continuously or intermittently within the genome, including both tandem and interspersed repeat sequences [4]. Tandem repeat sequences are localized in specific chromosomal regions, such as the centromere, telomere, and nucleolar organizer regions.

While interspersed repeat sequences typically evolve rapidly in plants, tandem repeat sequences are relatively conserved [5]. This high degree of conservation renders rDNA less susceptible to alterations during genetic processes; these repeat sequences are frequently utilized for chromosome recognition and karyotype analysis [6]. The rDNA loci mainly consist of rapidly differentiated intergenic spacer (IGS) regions, internal transcribed spacer (ITS) regions, and highly conserved coding regions. The highly conserved coding regions are reliable chromosomal markers and are extensively used in phylogenetic and karyotype studies [7,8]. During normal meiotic division of homologous chromosomes in plants, rDNA loci typically appear in pairs, with several pairs of homologous chromosomes each containing multiple pairs of rDNA loci. Abnormal segregation of homologous chromosomes can easily result in uneven chromosome division, leading to the failure of rDNA inheritance in offspring [9]. The presence of rDNA in pairs within plants indicates a more stable inheritance pattern. The distribution and quantity of rDNA on chromosomes can reflect the degree of interspecies differentiation, thereby influencing the assessment of evolutionary differences and genetic relationships among organisms.

In higher eukaryotes, the two most representative rDNA genes are 35S rDNA and 5S rDNA, which have been extensively utilized in the evolutionary studies of various plant species, including sugarcane, Arabidopsis, barley, and narcissus [10,11,12,13]. Research has revealed significant differences in the number and distribution of 35S rDNA and 5S rDNA sites among different species, with variations of thousands of copies within tandem arrays located at single or multiple chromosomal sites [14,15]. By leveraging these differences in copy number, cytogenetic analysis can elucidate the relationships among taxa and provide a framework for better understanding their evolution.

**Figure 1:**
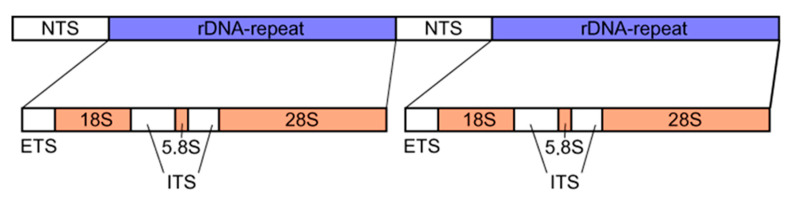
Structural diagram of 35S rDNA in higher plants [8].

The 35S rDNA locus is one of the most extensively studied chromosomal regions in eukaryotes. It typically resides on satellite chromosomes but can also be found on non-satellite chromosomes, albeit with a generally lower copy number [16]. The 35S rDNA locus is usually located at the secondary constriction sites of chromosomes, with a smaller proportion present at non-secondary constriction sites [17]. It is preferentially distributed on the short arm, mainly in the terminal regions of chromosomes. The 35S rDNA encodes the 28S, 5.8S, and 18S rRNA in sequence, primarily located in the nucleolar organizer regions (NORs), and is separated by IGSs, which include non-coding regions (NTSs) and external transcribed regions. The 28S, 5.8S, and 18S rRNA are separated by ITSs (Figure 1) [18]. All genes, except for the spacer regions, are highly conserved. The length of 35S rDNA repeat units varies among different plants, ranging from 7.8 to 18.5 kb, with copy numbers ranging from a few hundred to ten thousand. There are notable differences in the 35S rDNA gene between closely related species and even within the same individual of the same species. For instance, in different division phases of the same root tip in *Lolium*, the copy number and locus distribution of the 35S rDNA gene vary [19]. The genetic instability of the 35S rDNA gene has been documented in plants such as corn, barley, rice, sorghum, and ryegrass [20]. This instability is primarily reflected in differences in copy number, site distribution, and transposon activity in the early stages [19,21]. Recently, the human 35S rDNA gene has also been identified as a hotspot for DNA cleavage and recombination [22].

5S rDNA is present at one or several chromosomal positions throughout the genome, consisting of multiple tandem repeat sequences, primarily located near the centromere. Its copy number and loci vary with species evolution. 5S rDNA encodes 5S rRNA, which includes a 5S rRNA gene region transcribed by RNA polymerase III, a conserved 120 bp transcription region, and a variable non-transcriptional spacer region [23].

Research indicates that 5S rDNA near the centromere has a lower frequency of chromosomal translocation and deletion compared to 35S rDNA located at chromosomal ends, which is more prone to such events. The number of 5S rDNA copies in the genome is relatively stable, and therefore, 5S rDNA identified by fluorescence in situ hybridization (FISH) can represent the ploidy level of certain species.

### 1.2. Development and Utilization of 35S rDNA and 5S rDNA in Plants Based on FISH

FISH is a cutting-edge technology that has been steadily advancing in recent years. It primarily utilizes specific probes to precisely identify target DNA sequences on particular plant chromosomes under a microscope. It is a fast and reliable technology for studying the origin and evolution of polyploid species. In plant molecular cytogenetics, FISH technology, which can accurately locate DNA sequences, is widely used for constructing chromosome karyotypes and DNA physical maps to analyze genetic distances among closely related species. Therefore, FISH technology has significant potential and advantages for the recognition of plant chromosomes and karyotype construction. For example, chromosome genetic identification of sugarcane and wild hybrid offspring was conducted through FISH, and the authenticity of the hybrid offspring was detected by differential 5S rDNA analysis [24,25,26]. Kato et al. [27] successfully constructed maize chromosome recognition probes using repetitive sequence probes and employed FISH technology to distinguish 10 maize chromosomes. Meng et al. [28] used Oligo FISH to construct the chromosome karyotype of sugarcane, discovering chromosome recombination in *Saccharum spontaneum* and *Sorghum* based on the collinearity comparison of Oligo probe localization.

35S rDNA and 5S rDNA, as highly repetitive DNA sequence fragments, can serve as common molecular markers in eukaryotes. Due to their diverse and genetically stable marker sites within the genome and their resistance to external environmental conditions or gene expression, any tissue, organ, or cell of an individual can be used as a detection subject, offering advantages like short identification cycles and low economic costs. Consequently, they can be used as probes for FISH. The number of copies of important sequences of 35S rDNA and 5S rDNA on chromosomes can be determined by the fluorescence intensity of the probe. The number and position of 35S and 5S loci can be used to establish the chromosome karyotypes of each plant. Established chromosome karyotypes can systematically reflect changes in chromatin structure and quantity between plants, aiding in the exploration of relationships between species origin and evolution [29]. Wang et al. [30] used 35S rDNA to perform FISH on purple wheat, finding six 35S rDNA hybridization sites on the proximal part of the long arm of the chromosome, indicating that purple wheat is hexaploid. Zhang et al. [31] used FISH to physically locate 35S rDNA and 5S rDNA on the somatic metaphase chromosomes of three wild rose species. They found that 35S rDNA was located at the short arms of a pair of heterologous homologous chromosomes at the centromere, and the number and distribution patterns of the three materials were relatively consistent. However, there were differences in the quantity and location of 5S rDNA among these three materials, with one pair of loci on the multi-bracted rose and two pairs of loci each on the Sichuan Yunnan rose and the cherry blossom, both located near the centromere of the chromosome long arm, which can be used for identifying related germplasm. Xu et al. [17] used 35S rDNA-FISH to study the chromosome localization of crops such as common wheat, hard cluster wheat, cluster wheat, and barley. They found that the chromosome secondary constriction regions of these materials all have 35S rDNA sites, confirming that 35S rDNA is not only located on the satellite chromosome but also partially on the non-satellite chromosome. For the first time, FISH technology was used to locate the wheat goosegrass 35S rDNA on the short arm of the chromosome. Moreover, Jiang et al. [23] compared the number and positional characteristics of 35S rDNA and 5S rDNA on the chromosomes of Chrysanthemum and its related genera using FISH, analyzing the phylogenetic relationships between genera and species. Therefore, rDNA as a stable chromosome marker has been widely used in chromosome identification and karyotype construction using FISH technology.

## 2. Research on the Phylogenetic Relationship and Evolutionary Relationship of rDNA in Sugarcane

*Saccharum* spp. are primarily distributed in tropical and subtropical regions. As the world’s most important sugar crop, sugarcane accounts for over 90% of annual sugar production [32]. Sugarcane is also one of the most significant bioenergy crops [33], with electricity and ethanol derived from sugarcane providing a green energy source that can replace fossil fuels. The taxonomy of sugarcane is complex and controversial [34]. The genus *Saccharum* was traditionally divided into four cultivated species and two wild species, although this is now questioned (references). The four cultivated species are *S. officinarum*, *S. sinense*, *S. barberi*, and *S. edule*, while the two wild species are *S. spontaneum* and *S. robustum* [35]. *S. sinense* and *S. barberi* are considered natural hybrids of *S. officinarum* and *S. spontaneum*, indicating that the lineage of cultivated sugarcane varieties primarily originates from *S. officinarum* and *S. spontaneum* [36,37]. Modern commercial sugarcane varieties are mostly obtained through hybridization between varieties; consequently, long-term interspecific hybridization has limited the innovation of sugarcane materials. In recent years, breeders have increasingly utilized wild germplasm resources from sugarcane-related genera, aiming to develop a breakthrough in breeding better sugarcane varieties. *Tripidium arundinaceum* is a plant related to sugarcane; before 2019, it was called *Erianthus arundinaceus* and classified under *Erianthus*, but through analysis of chloroplasts and a gene locus phylogeny, it was ultimately classified under *Tripidium* [38]. It has been identified and used by sugarcane breeders due to its strong growth potential, excellent tillering ability, robust rooting capability, and strong resistance. Sugarcane breeders worldwide continue to undertake inter-generic distant hybridization, aiming to introduce superior genes of wild sugarcane-related species into sugarcane, enrich its genetic background, and create superior parent varieties with high sugar content, high yield, and stress resistance. In sugarcane distant hybridization, the identification of hybrid authenticity and chromosomal genetic research are crucial for genetic progress and subsequent studies, where 35S rDNA and 5S rDNA play significant roles. Compared to corn, rice, wheat, and sorghum in the Poaceae family, sugarcane exhibits a diverse ploidy, significantly complicating genetics research. Utilizing 35S rDNA and 5S rDNA as probe markers, FISH technology can elucidate the transmission modes and some characteristics of chromosomes in various sugarcane species and their related genera [39]. This provides a foundation for the more effective development and utilization of wild sugarcane germplasm resources in breeding programs through cytogenetics [40].

### 2.1. Study on the Ploidy Relationship and Distribution of Sugarcane Using 35S rDNA and 5S rDNA

In the sugarcane genus, 35S rDNA and 5S rDNA of *S. officinarum* are located at the ends of chromosomes, whereas in *S. spontaneum*, they are embedded between chromosome arms and near the centromere [41]. This may result from extensive chromosomal rearrangement during sugarcane evolution, causing these loci to shift on chromosomes. The most notable application of rDNA localization in the sugarcane genus has been to determine the chromosomal base of *S. officinarum*, *S. spontaneum*, and *S. robustum*. The localization of rDNA in sugarcane was first discovered and applied by D’hont et al. [24]. They showed that 35S rDNA and 5S rDNA can be labeled at different positions on the same set of chromosomes. Based on the number of FISH sites for 35S rDNA and 5S rDNA in chromosomes, it was inferred that the chromosome base number for *S. officinarum* and *S. robustum* is x = 10, while for *S. spontaneum*, it is x = 8. Additionally, Li et al. [42] used 5S rDNA to identify that the chromosome base number for different ploidy levels of *S. spontaneum* is x = 8. Ha et al. [43] identified a tetraploid *S. spontaneum* (2*n* = 32) with four 5S rDNA and four 35S rDNA loci by using FISH technology, confirming that the chromosome base number is also x = 8, while FISH mapping of 35S rDNA and 5S rDNA in sugarcane chromosomes has been effective in determining germplasm ploidy; the signal site for 5S rDNA is generally more stable than that for 35S rDNA. The number of signal sites for 5S rDNA usually corresponds to the chromosome ploidy, and the signal intensity among sites is consistent. This stability may be because 5S rDNA is located near the centromere of the chromosomes, resulting in a slower evolution rate and smaller copy number differences between chromosomes. In contrast, the number of 35S rDNA signals may not always correspond to chromosome ploidy in some sugarcane materials. For example, Huang et al. [9] detected only five and seven 35S rDNA FISH signals in a decaploid *S. spontaneum* and an octoploid *S. officinarum*, respectively. Additionally, irregular site reduction of 35S rDNA was observed in different *S. spontaneum* chromosomes, possibly due to the rapid evolution of 35S rDNA and the low copy number in some chromosomes, making visual FISH signals difficult to detect. Thus, FISH localization of 5S rDNA is more reliable for determining chromosome ploidy in sugarcane than 35S rDNA.

In 1995, D’hont et al. [10] used the 35S rDNA clone pTa71 of wheat as a probe to study the 35S rDNA-FISH of *T. arundinaceum*. They found six hybridization site signals at the chromosome ends, confirming that the chromosome number was 2*n* = 60 and the base number x = 10. Experiments by Wu et al. [44] revealed that both 35S rDNA and 5S rDNA had six signal sites on the chromosomes of the *T. arundinaceum* clones Hainan 92-77 and Hainan 92-105, although their localization positions varied. The six 35S rDNA sites were located at the ends of the chromosomes’ short arms, but differed in copy number. The six 5S rDNA sites were all near the centromere of the long arm of the chromosome, with no difference in copy number. Additionally, Besse et al. [45] used 35S rDNA as a probe for FISH localization in *Erianthus* Michx., finding that *E.* Michx. with 2*n* = 20 and 2*n* = 40 chromosomes had two and four 35S rDNA sites, respectively. This confirmed that the chromosome base number of *E.* Michx. was x = 10. Furthermore, Wu et al. [44] verified that both 35S rDNA and 5S rDNA had six sites in two *T. arundinaceum* (2*n* = 6x = 60) clones, reinforcing that the chromosome base of *T. arundinaceum* was x = 10, but they noted differences in the copy numbers of the six 35S rDNA sites. Moreover, Huang et al. [46] found that the positions of 35S rDNA and 5S rDNA in metaphase cell chromosomes of *T. arundinaceum* were distinct: 35S rDNA was located at the chromosome ends, while 5S rDNA was near the centromeres [47].

Polyploidization is a natural phenomenon in plant evolution, and polyploid plants are common, with important examples including wheat, daffodils, strawberries, and sugarcane. The number of 35S rDNA and 5S rDNA loci is often positively correlated with the ploidy level of plants, allowing the determination of plant ploidy based on the number of these loci. During chromosomal ploidy evolution, each additional set of chromosomes results in an increase of one corresponding 35S rDNA and 5S rDNA locus. The number and location of 35S rDNA and 5S rDNA loci have become important reference criteria for determining the origin of polyploid chromosomes. A review of the published literature reveals a wide range in ploidy levels in sugarcane-related genera and Poaceae plants in studies based on the number of 35S rDNA and 5S rDNA loci (Table 1). The results indicate that plants of the same genus and species perhaps have different levels of ploidy. The number of 5S rDNA and 35S rDNA loci can indicate the ploidy of the plant. 5S rDNA is mainly located in the centromere region of the chromosome, while 35S rDNA loci are mainly located at the end of the chromosome.

**Table 1:** Number of 35S rDNA and 5S rDNA loci in sugarcane, sugarcane-related genera, and Poaceae plants.

Species	5S Loci Num	5S Position	35S Loci Num	35S Position	2*n*	Ploidy
*S. spontaneum* [48]	14	interstitial	10	interstitial	112	14
*S. spontaneum* [48]	12	interstitial	12	interstitial	96	12
*S. spontaneum* [48]	10	interstitial	10	interstitial	80	10
*S. spontaneum* [48]	8	interstitial	8	interstitial	64	8
*S. spontaneum* [43]	4	(peri-)centromeric	4	satellite	32	4
*S. officinarum* [48]	8	interstitial	8	(sub-)terminal or satellite	80	8
*S. robustum* [24]	6	interstitial	6	(sub-)terminal or satellite	60	6
*S. robustum* [24]	8	interstitial	8	(sub-)terminal or satellite	80	8
*S. arundinaceum* [44]	6	interstitial	6	(sub-)terminal or satellite	60	6
*Triticum aestivum* [49]	2	(sub-)terminal	6	(sub-)terminal	42	6
*Oryza glaberrima* [50]	2	interstitial	2	(peri-)centromeric	24	2
*Zea mays* [51]	2	(sub-)terminal	2	satellite	20	2
*Sorghum bicolor* [52]	/	/	2	(peri-)centromeric	20	2

### 2.2. Application of 35S rDNA and 5S rDNA in the Study of Sugarcane Evolution and Genetic Relationship

For species with a large number of chromosomes and similar morphology, conventional karyotype analysis may be accurate [53]. The localization of 35S rDNA and 5S rDNA on different chromosomes undoubtedly supplements and assists traditional karyotype analysis. Unlike 35S rDNA, the location of the 5S rDNA gene on chromosomes is notably associated with the nucleolar organizer region, and its distribution is more diverse [54]. The 35S rDNA consists of coding regions 18S, 5.8S, 28S rDNA, ITS, and IGS. The 5.8S rDNA divides ITS1 and ITS2 fragments. The transcripts of ITS1 and ITS2 are cleaved during rRNA processing but play an important role in rRNA maturation [55]. Although 35S rDNA is highly conserved, there are variations in ITSs and IGSs [56]. In recent years, the ITS has been recognized as a DNA fragment conserved in length during the evolution of the nuclear genome, but with significant nucleotide sequence variation, rapid evolution, high stability, and ease of sequencing. This characteristic makes its sequence valuable for determining the genetic relationships between species through sequencing [55,56,57,58,59].

Among the sugarcane-related genera, *Erianthus* Michx., *Miscanthus Andersson*, *Sclerostachya* (Hach.) *A. camus*, and *Narenga* Bor are important wild germplasm resources and are classified as the Saccharum complex [42]. Researchers have determined the ITS region of ribosomal DNA and the gene sequence of 5.8S rDNA in 13 species of *Saccharum* and its related genera. ITS sequence length in sugarcane and its related genera ranges from 589 to 591 bp, and the length of 5.8S rDNA is 164 bp. The results from constructing a phylogenetic tree indicate that Zhejiang chewing cane belongs to *S. officinarum*, and that the genetic relationship between *Narenga Bor* and *E.* Michx. is relatively close, while *T. arundinaceum* does not belong to *Saccharum* [60]. Concurrently, Liu et al. [61] constructed an evolutionary tree using ITS sequences, revealing that *M.* Andersson and *Triarrhena* have the closest genetic relationship with *Saccharum*, followed by *Narenga Bor* and *E.* Michx. In contrast, Imperata has a distant genetic relationship with *Saccharum*. Additionally, *T. arundinaceum* was classified under *E.* Michx., and *Triarrhena* was classified under *M.* Andersson, consistent with results obtained by Hu et al. [62] through IGS sequence analysis. Lee et al. [63] constructed phylogenetic relationships between different ploidy *S. spontaneum* sites by analyzing ITS sequences of 35S rDNA and NTS sequences of 5S rDNA. The study inferred that the genetic relationship between dodecaploid and nonuple material of *S. spontaneum* is the closest, followed by the relationship between decaploid and octaploid material of *S. spontaneum*. These findings demonstrate the value in using ITS sequences to determine phylogenetic relationships between species and genera. Moreover, the mutation rate of the IGS is relatively fast, but its function is conserved, including transcription start points, transcription end sites, and various regulatory factors [64,65]. The physical localization results of repeated sequences from multiple species indicate the diversity of the 35S rDNA-FISH site, suggesting that the 35S rDNA gene has undergone extensive evolution throughout the entire genome.

### 2.3. Application of 35S rDNA and 5S rDNA in Chromosome Composition and Genetics

Currently, utilizing bioinformatics technology allows for the comparison of the localization of 35S rDNA and 5S rDNA on various plant chromosomes. By downloading the genomes of sorghum and maize from NCBI (https://www.ncbi.nlm.nih.gov/, accessed on 30 June 2021) and comparing the sequences of 35S rDNA and 5S rDNA, it was found that the sequences of 35S rDNA and 5S rDNA were highly consistent with those of chromosomes 5 and 9 in sorghum and chromosomes 6 and 2 in maize, respectively [66,67]. Moreover, Yu et al. [41] used maize chromosomes to design probes and, for the first time, differentiated chromosomes 1–10 of *T. arundinaceum*. Cytological analysis and FISH experiments with 5S rDNA and 35S rDNA confirmed that 5S rDNA and 35S rDNA are located on chromosomes 5 and 6 of *T. arundinaceum*, respectively. These results indicate that the repeat sequences of 35S rDNA and 5S rDNA have a high degree of overlap with the sequence of a certain chromosome in various species, and 35S rDNA and 5S rDNA can be used to determine the composition of certain plant chromosomes. In addition, the genetic pattern of plant chromosomes can be determined by the number of loci located in offspring and parents using 35S rDNA FISH and 5S rDNA FISH. Through FISH, it was found that there were eight loci of 35S rDNA in the *S. officinarum* clone Badila (2*n* = 80), six loci of 35S rDNA in the *T. arundinaceum* clone HN92-77 (2*n* = 60), and seven loci of 35S rDNA in the clone Yacheng 95-41, which was a hybrid between *S. officinarum* and *T. arundinaceum*, including four from *S. officinarum* and three from *T. arundinaceum*. GISH experiments have shown that there was *n* + *n* chromosome transmission in hybrids between *S. officinarum* and *T. arundinaceum*. FISH experiments using 35S rDNA also confirmed that the inheritance in these hybrids was *n* + *n*. Meanwhile, Li investigated the problem of F_1_ pollen sterility in the hybrid offspring of *S. officinarum* and *T. arundinaceum* by using 35S rDNA and 5S rDNA. During meiosis, the pollen mother cells of F_1_ have six 35S rDNA sites and six 5S rDNA sites. During meiosis II, one 35S rDNA site was lost, while during meiosis, the number of 5S rDNA sites in each cell varied, and abnormalities were found during the tetrad stage. This experiment provides clear and intuitive experimental evidence for F_1_ pollen sterility [68].

## 3. Outlook

rRNA, as a repetitive sequence distributed in clusters at one or several loci with copy numbers ranging from 500 to 40,000, is the most studied genetic unit in the plant genome. Its chromosomal localization provides stable and effective markers for karyotype analysis, making it a crucial tool for constructing molecular karyotypes and investigating genome structure, function, and evolution [23]. rDNA has been extensively physically localized in the genomes of many important model and economic crops, providing vital information for species chromosome recognition, genome structure analysis, physical map construction, and species phylogenetic research [69,70,71]. As mentioned earlier, there have been numerous reports on the localization of 35S rDNA and 5S rDNA on sugarcane chromosomes. However, due to the limited distribution of rDNA repeat sequences on the chromosomes of species, only some chromosomes contain these sequences, and their loci are unstable [72]. Therefore, the role of rDNA as a chromosome recognition marker has certain limitations. Cultivated sugarcane plants are unusual among leading crops; they are polyploid interspecific hybrids, with singularly complex genomes, and have a complex chromosome composition, and there is no significant difference in morphology and size between adjacent chromosomes. Additionally, the varying degrees of chromosome condensation during mitosis can cause interference and positioning errors, leading to inaccurate identification of individual chromosomes. For instance, the number of chromosomes in *T. arundinaceum* is relatively large, and their morphology is small. The lengths of chromosomes 6, 7, and 8 in *T. arundinaceum* are very similar, making it challenging to accurately pair homologous chromosomes and identify and arrange different chromosomes using only conventional chromosome karyotype analysis methods. Without specific bands or recognition sites, it is impossible to accurately identify all chromosomes in the set of chromosomes of *T. arundinaceum*. To accurately identify sugarcane chromosomes, researchers have attempted to use rDNA-like repetitive sequences as probes, combined with rDNA for localization, or to further improve FISH resolution. Using BAC clones or SSR markers as probes for FISH detection and referring to the linkage and physical maps of related plant groups can aid in the identification of individual chromosomes [73,74,75,76].

The application of rDNA-specific sequences in ploidy identification, evolution, and phylogenetic studies in sugarcane taxa has been previously discussed. Future research should focus on a comprehensive understanding of ITS sequences. When using these sequences for phylogenetic research in related taxa, it is essential to consider their polymorphisms within individuals and to evaluate their applicability promptly. Additionally, to construct a phylogenetic tree that closely reflects reality, there is an increasing trend of integrating multiple DNA-specific sequences. This includes the use of chloroplast DNA fragments and other nuclear DNA fragments in combination with rDNA to study the phylogenetic relationships of sugarcane [74]. Specifically, with the advancements in determining the complete genome sequence of sugarcane and the identification and isolation of genome repeat sequences, future analyses can leverage published genome information to examine the distribution and sequence variations of rDNA. This approach not only facilitates a more comprehensive exploration of sugarcane phylogeny but also provides a foundation for identifying chromosomally specific markers and obtaining DNA probes for specific chromosomes. In summary, the comparative localization of 35S rDNA and 5S rDNA on sugarcane chromosomes and their application in phylogenetic studies will become a crucial direction for future sugarcane chromosome recognition and phylogenetic research. This will be achieved by exploring the distribution and sequence changes of rDNA throughout the genome in specific groups or by combining rDNA with other specific sequences.

Future research should focus on combining multi-omics data with high-resolution technology to deepen the application of rDNA in the sugarcane genome. For example, using third-generation sequencing and optical mapping technology, the structural variations and distribution patterns of rDNA units can be accurately analyzed; developing a multi-color FISH system that combines rDNA with single-copy sequences, BAC clones, or SSR markers can help establish a more reliable chromosome recognition system. In terms of phylogenetics, rDNA should be integrated with low-copy nuclear genes and chloroplast genome data to construct a multi-gene evolutionary tree, in order to improve node support and topological reliability. In addition, chromosome-specific probes based on completed genome design and their co-segregation analysis in hybrid populations will also provide a new path for sugarcane chromosome identification and evolutionary research. Through these strategies, the limitations of current rDNA markers can be overcome, and the research on sugarcane cell genetics and phylogenetics will be promoted to a more refined and integrated stage.

## Data Availability

The data are contained in this article.

## References

[1] Sun Y., Skinner D.Z., Liang G.H., Hulbert S.H. (1994). Phylogenetic analysis of *Sorghum* and related taxa using internal transcribed spacers of nuclear ribosomal DNA. Theor. Appl. Genet..

[2] Sang T., Crawford D.J., Stuessy T.F. (1995). Documentation of reticulate evolution in peonies (*Paeonia*) using internal transcribed spacer sequences of nuclear ribosomal DNA: Implications for biogeography and concerted evolution. Proc. Natl. Acad. Sci. USA.

[3] Castilho A., Heslop-Harrison J.S. (1995). Physical mapping of 5S and 18S-25S rDNA and repetitive DNA sequences in *Aegilops umbellulata*. Genome.

[4] Kubis S., Schmidt T., Heslopharrison, Pat J.S. (1998). Repetitive DNA Elements as a Major Component of Plant Genomes. Ann. Bot..

[5] Flavell R.B., Bennett M.D., Smith J.B., Smith D.B. (1974). Genome size and the proportion of repeated nucleotide sequence DNA in plants. Biochem. Genet..

[6] Koornneef M., Fransz P., Jong H.D.J. (2003). Cytogenetic tools for Arabidopsis thaliana. Chromosome Res..

[7] Badaeva E.D., Friebe B., Gill B.S. (1996). Genome differentiation in Aegilops. 1. Distribution of highly repetitive DNA sequences on chromosomes of diploid species. Genome.

[8] Huang Y. (2014). Chromosome Inheritance for the BC_1_ Progeny of Sugarcane and *Erianthus arundinaceus* Based on 45S rDNA-FISH and GISH. Master’s Thesis.

[9] Huang Y., Yu F., Li X., Luo L., Wu J., Yang Y., Deng Z., Chen R., Zhang M. (2017). Comparative genetic analysis of the 45S rDNA intergenic spacers from three Saccharum species. PLoS ONE.

[10] D’Hont A., Rao P.S., Feldmann P., Grivet L., Islam-Faridi N., Taylor P., Glaszmann J.C. (1995). Identification and characterisation of sugarcane intergeneric hybrids *Saccharum officinarum* × *Erianthus arundinaceus*, with molecular markers and DNA in situ hybridization. Theor. Appl. Genet..

[11] Fransz P., Armstrong S., Alonso-Blanco C., Fischer T.C., Torres-Ruiz R.A., Jones G. (1998). Cytogenetics for the model system *Arabidopsis thaliana*. Plant J. Cell Mol. Biol..

[12] Wu J., Zhang Z., Lv L. (2008). Chromosome Localization of 45S rDNA and 5S rDNA in Chinese Narcissus by Fluorescence in situ Hybridization. Chin. J. Trop. Crops.

[13] Liao J., Yang R., Zhou Y., Tsujimoto H. (2007). FISH analysis of 45S rDNA and 5S rDNA genes in *Triticum polonicum* L. and *T. turgidum* L. cv. Ailanmai. Hereditas.

[14] Wang Y., Wang C., Jiang Y., Katz L.A., Gao F., Yan Y. (2019). Further analyses of variation of ribosome DNA copy number and polymorphism in ciliates provide insights relevant to studies of both molecular ecology and phylogeny. Sci. China. Life Sci..

[15] Lutzoni F., Wagner P., Reeb V., Zoller S. (2000). Integrating ambiguously aligned regions of DNA sequences in phylogenetic analyses without violating positional homology. Syst. Biol..

[16] Waminal N.E., Kim H.H. (2012). Dual-color FISH karyotype and rDNA distribution analyses on four Cucurbitaceae species. Hortic. Environ. Biotechnol..

[17] Xu C., Bie T., Wang C., Zhou B., Chen P. (2007). Distribution of 45S rDNA sequence on chromosomes of *Triticum aestivum* and its relative species. Hereditas.

[18] Baldwin B.G., Markos S. (1998). Phylogenetic utility of the external transcribed spacer (ETS) of 18S-26S rDNA: Congruence of ETS and ITS trees of *Calycadenia* (Compositae). Mol. Phylogenetics Evol..

[19] Thomas H.M., Harper J.A., Meredith M.R., Morgan W.G., Thomas I.D., Timms E., King I.P. (1996). Comparison of ribosomal DNA sites in Lolium species by fluorescence in situ hybridization. Chromosome Res..

[20] Huang M., Li H., Zhang L., Gao F., Wang P., Hu Y., Yan S., Zhao L., Zhang Q., Tan J. (2012). Plant 45S rDNA clusters are fragile sites and their instability is associated with epigenetic alterations. PLoS ONE.

[21] Schubert I., Wobus U. (1985). In situ hybridization confirms jumping nucleolus organizing regions in *Allium*. Chromosoma.

[22] Stults D.M., Killen M.W., Williamson E.P., Hourigan J.S., Vargas H.D., Arnold S.M., Moscow J.A., Pierce A.J. (2009). Human rRNA gene clusters are recombinational hotspots in cancer. Cancer Res..

[23] Jiang J., Chen S., Zhang F., Qi F., Guan X. (2015). Localization of 45S and 5S rDNA sites and karyotype of Chrysanthemum and its related genera by fluorescent in situ hybridization. Biochem. Syst..

[24] D’Hont A., Ison D., Alix K., Roux C., Glaszmann J.C. (1998). Determination of basic chromosome numbers in the genus *Saccharum* by physical mapping of ribosomal RNA genes. Genome Biol. Evol..

[25] Piperidis G., Christopher M.J., Carroll B.J., Berding N., D’Hont A. (2000). Molecular contribution to selection of intergeneric hybrids between sugarcane and the wild species *Erianthus arundinaceus*. Genome.

[26] Cuadrado A., Acevedo R., Moreno Díaz de la Espina S., Jouve N., de la Torre C. (2004). Genome remodelling in three modern *S. officinarum* × *S. spontaneum* sugarcane cultivars. J. Exp. Bot..

[27] Kato A., Lamb J.C., Birchler J.A. (2004). Chromosome painting using repetitive DNA sequences as probes for somatic chromosome identification in maize. Proc. Natl. Acad. Sci. USA.

[28] Meng Z., Zhang Z., Yan T., Lin Q., Wang Y., Huang W., Huang Y., Li Z., Yu Q., Wang J. (2018). Comprehensively Characterizing the Cytological Features of *Saccharum spontaneum* by the Development of a Complete Set of Chromosome-Specific Oligo Probes. Front. Plant Sci..

[29] Zhang L., Yang X., Tian L., Chen L., Yu W. (2016). Identification of peanut (*Arachis hypogaea*) chromosomes using a fluorescence in situ hybridization system reveals multiple hybridization events during tetraploid peanut formation. New Phytol..

[30] Wang Y., Qin W., Hao L., Wang X., Ma Y., Liu Y. (2020). Analysis of karyotype and 45S rDNA locus of purple grain wheat. Biotechnology.

[31] Zhang T., Jian H., Tian M., Wang Q., Zhang H., Yan H., Qiu X., Tang K. (2014). Physical Location of 45S rDNA and 5S rDNA in the Genomes of Three Wild Rose Species. Acta Hortic. Sin..

[32] Wang J., Roe B., Macmil S., Yu Q., Murray J.E., Tang H., Chen C., Najar F., Wiley G., Bowers J. (2010). Microcollinearity between autopolyploid sugarcane and diploid sorghum genomes. BMC Genom..

[33] Kole C. (2007). Pulses, Sugar and Tuber Crops.

[34] Evans D.L., Joshi S.V. (2016). Complete chloroplast genomes of *Saccharum spontaneum*, *Saccharum officinarum* and *Miscanthus floridulus* (Panicoideae: Andropogoneae) reveal the plastid view on sugarcane origins. Syst. Biodivers..

[35] Amalraj V.A., Balasundaram N. (2006). On the Taxonomy of the Members of ‘Saccharum Complex’. Genet. Resour. Crop Evol..

[36] Ming R. (2006). Sugarcane improvement through breeding and biotechnology. Plant Breed. Rev..

[37] Zhang J., Zhang Q., Li L., Tang H., Zhang Q., Chen Y., Arrow J., Zhang X., Wang A., Miao C. (2019). Recent polyploidization events in three Saccharum founding species. Plant Biotechnol. J..

[38] Lloyd Evans D., Joshi S.V., Wang J. (2019). Whole chloroplast genome and gene locus phylogenies reveal the taxonomic placement and relationship of *Tripidium* (Panicoideae: Andropogoneae) to sugarcane. BMC Evol. Biol..

[39] Pachakkil B., Terajima Y., Ohmido N., Ebina M., Irei S., Hayashi H., Takagi H. (2019). Cytogenetic and agronomic characterization of intergeneric hybrids between *Saccharum* spp. hybrid and *Erianthus arundinaceus*. Sci. Rep..

[40] Pan Y.B., Burner D.M., Legendre B.L. (2000). An assessment of the phylogenetic relationship among sugarcane and related taxa based on the nucleotide sequence of 5S rRNA intergenic spacers. Genetica.

[41] Yu F., Chai J., Li X., Yu Z., Yang R., Ding X., Wang Q., Wu J., Yang X., Deng Z. (2021). Chromosomal Characterization of *Tripidium arundinaceum* Revealed by Oligo-FISH. Int. J. Mol. Sci..

[42] Li S., Wang X., Yang Q. (2018). The Ploidy Identification of *Saccharum spontaneum* Collected from Myanmar Based on 5S rDNA-FISH Localization. Mol. Plant Breed..

[43] Ha S., Moore P.H., Heinz D., Kato S., Ohmido N., Fukui K. (1999). Quantitative chromosome map of the polyploid *Saccharum spontaneum* by multicolor fluorescence in situ hybridization and imaging methods. Plant Mol. Biol..

[44] Wu J., Huang Y., Wang Q., Ling Q., Deng Z., Li Q., Chen R. (2018). Physical Mapping of rDNA on the Chromosomes of *Erianthus arundinaceus* and Determination of Basic Chromosome Number. Sugarcane Canesugar.

[45] Besse P., Taylor G., Carroll B., Berding N., Burner D., Mcintyre C.L. (1998). Assessing genetic diversity in a sugarcane germplasm collection using an automated AFLP analysis. Genetica.

[46] Huang Y., Wu J., Wang P., Lin Y., Fu C., Deng Z., Wang Q., Li Q., Chen R., Zhang M. (2015). Characterization of Chromosome Inheritance of the Intergeneric BC_2_ and BC_3_ Progeny between *Saccharum* spp. and *Erianthus arundinaceus*. PLoS ONE.

[47] Wu J., Huang Y., Lin Y., Fu C., Liu S., Deng Z., Li Q., Huang Z., Chen R., Zhang M. (2014). Unexpected inheritance pattern of *Erianthus arundinaceus* chromosomes in the intergeneric progeny between *Saccharum* spp. and *Erianthus arundinaceus*. PLoS ONE.

[48] D’Hont A. (2005). Unraveling the genome structure of polyploids using FISH and GISH; examples of sugarcane and banana. Cytogenet. Genome Res..

[49] Appels R., Gerlach W.L., Dennis E.S., Swift H., Peacock W.J. (1980). Molecular and chromosomal organization of DNA sequences coding for the ribosomal RNAs in cereals. Chromosoma.

[50] Ohmido N., Fukui K. (1995). Cytological studies of African cultivated rice, Oryza glaberrima. TAG. Theor. Appl. genetics. Theor. Und Angew. Genet..

[51] Han Y.H., Li L.J., Song Y.C., Li Z.Y., Xiong Z.Y., Li D.Y. (2002). Physical mapping of the 5S and 45S rDNA in teosintes. Hereditas.

[52] Sang Y., Liang G.H. (2000). Comparative physical mapping of the 18S-5.8S-26S rDNA in three sorghum species. Genome.

[53] Maoyin S. (2013). Physical Mapping of the 45S and 5S rDNA of Cultivated Buckwheat. J. Plant Genet. Resour..

[54] Mizuochi H., Marasek A., Okazaki K. (2007). Molecular cloning of Tulipa fosteriana rDNA and subsequent FISH analysis yields cytogenetic organization of 5S rDNA and 45S rDNA in *T. gesneriana* and *T. fosteriana*. Euphytica.

[55] Wang J., Wang W., Chen J. (1999). Application of ITS sequences of nuclear rDNA in phylogenetic and evolutionary studies of angiosperms. J. Syst. Evol..

[56] Poczai P., Hyvönen J. (2010). Nuclear ribosomal spacer regions in plant phylogenetics: Problems and prospects. Mol. Biol. Rep..

[57] Wang X., Deng Z., Hong D. (1998). The Systematic Position of Beesia: Evidence from ITS (nrDNA) Sequence Analysis. Acta Phytotaxon. Sin..

[58] Ainouche M.L., Bayer R.J. (1997). On the origins of the tetraploid *Bromus* species (section *Bromus*, Poaceae): Insights from internal transcribed spacer sequences of nuclear ribosomal DNA. Genome.

[59] Sanderson M.J., Baldwin B.G., Porter J.M., Wojciechowski M.F., Campbell C.S. (1995). The ITS Region of Nuclear Ribosomal DNA: A Valuable Source of Evidence on Angiosperm Phylogeny. Ann. Mo. Bot. Gard..

[60] Chen H., Fan Y., Xiangyu J., Cai Q., Zhang Y. (2003). Phylogenetic Relationships of Saccharum and Related Species Inferred from Sequence Analysis of the nrDNA ITS Region. Acta Agron. Sin..

[61] Hodkinson T.R., Chase M.W., Lledó M.D., Salamin N., Renvoize S.A. (2002). Phylogenetics of *Miscanthus*, *Saccharum* and related genera (Saccharinae, Andropogoneae, Poaceae) based on DNA sequences from ITS nuclear ribosomal DNA and plastid *trnL* intron and *trnL-F* intergenic spacers. J. Plant Res..

[62] Hu X.G., Yu F., Huang Y.J., Sun L., Li X.T., Yang S., Chen K., Huang F., Zeng K., Zhang M.Q. (2019). Characterization analysis of the 35S rDNA intergenic spacers in *Erianthus arundinaceus*. Gene.

[63] Lee H.I., Younis A., Hwang Y.J., Kang Y.I., Lim K.B. (2014). Molecular cytogenetic analysis and phylogenetic relationship of 5S and 45S ribosomal DNA in sinomartagon *Lilium* species by fluorescence in situ hybridization (FISH). Hortic. Environ. Biotechnol..

[64] Gruendler P., Unfried I., Pascher K., Schweizer D. (1991). rDNA intergenic region from *Arabidopsis thaliana*. Structural analysis, intraspecific variation and functional implications. J. Mol. Biol..

[65] Volkov R.A., Bachmair A., Panchuk I.I., Kostyshyn S.S., Schweizer D. (1999). 25S-18S rDNA intergenic spacer of *Nicotiana sylvestris* (*Solanaceae*): Primary and secondary structure analysis. Plant Syst. Evol..

[66] Chai J., Luo L., Yu Z., Lei J., Zhang M., Deng Z. (2022). Repetitive Sequence Barcode Probe for Karyotype Analysis in *Tripidium arundinaceum*. Int. J. Mol. Sci..

[67] Mahelka V., Kopecky D., Baum B.R. (2013). Contrasting patterns of evolution of 45S and 5S rDNA families uncover new aspects in the genome constitution of the agronomically important grass *Thinopyrum intermedium* (Triticeae). Mol. Biol. Evol..

[68] Li X.T., Huang F., Chai J., Wang Q.S., Yu F., Huang Y.J., Wu J.Y., Wang Q.N., Xu L.N., Zhang M.Q. (2021). Chromosome behavior during meiosis in pollen mother cells from *Saccharum officinarum* x *Erianthus arundinaceus* F_1_ hybrids. BMC Plant Biol..

[69] Falistocco E., Passeri V., Marconi G. (2007). Investigations of 5S rDNA of Vitis vinifera L.: Sequence analysis and physical mapping. Genome.

[70] Lan T., Liu B., Dong F., Chen R., Li X., Chen C. (2007). Multicolor FISH analysis of rDNA and telomere on spinach. Hereditas.

[71] Jiang J., Gill B.S. (1994). New 18S.26S ribosomal RNA gene loci: Chromosomal landmarks for the evolution of polyploid wheats. Chromosoma.

[72] Kulak S., Hasterok R., Maluszynska J. (2002). Karyotyping of *Brassica amphidiploids* using 5S and 25S rDNA as chromosome markers. Hereditas.

[73] Moraes A.P., Mirkov T.E., Guerra M. (2008). Mapping the chromosomes of *Poncirus trifoliata* Raf. by BAC-FISH. Cytogenet. Genome Res..

[74] Morton C.M. (2009). Phylogenetic relationships of the Aurantioideae (Rutaceae) based on the nuclear ribosomal DNA ITS region and three noncoding chloroplast DNA regions, *atpB-rbcL* spacer, *rps16*, and *trnL*-*trnF*. Org. Divers. Evol..

[75] Wai C.M., Ming R., Moore P.H., Paull R.E., Yu Q. (2010). Development of Chromosome-specific Cytogenetic Markers and Merging of Linkage Fragments in *Papaya*. Trop. Plant Biol..

[76] Wu J., Zhang M., Liu J., Huang Y., Xu L., Deng Z., Zhao X. (2022). Efficient Anchoring of *Erianthus arundinaceus* Chromatin Introgressed into Sugarcane by Specific Molecular Markers. Int. J. Mol. Sci..

